# Life Expectancy after Surgery for Ascending Aortic Aneurysm

**DOI:** 10.3390/jcm9030615

**Published:** 2020-02-25

**Authors:** Daniel Hernandez-Vaquero, Jacobo Silva, Alain Escalera, Rubén Álvarez-Cabo, Carlos Morales, Rocío Díaz, Pablo Avanzas, Cesar Moris, Isaac Pascual

**Affiliations:** 1 Cardiac Surgery Department, Central University Hospital of Asturias, 33011 Oviedo, Spain; jsilva8252@yahoo.es (J.S.); alain_2623@hotmail.com (A.E.); motocarlos24@yahoo.com (C.M.); diazmendezro@gmail.com (R.D.); 2 Instituto de Investigación Sanitaria del Principado de Asturias, 33011 Oviedo, Spain; avanzas@gmail.com (P.A.); cesarmoris@gmail.com (C.M.); ipascua@live.com (I.P.); 3 Department of Surgery, University of Oviedo, 33011 Oviedo, Spain; 4 Department of Cardiology, Central University Hospital of Asturias, 33011 Oviedo, Spain; 5 Department of Medicine, University of Oviedo, 33011 Oviedo, Spain

**Keywords:** ascending aortic aneurysm, ascending aortic replacement, life expectancy

## Abstract

**Introduction**: The life expectancy of patients who undergo ascending aortic replacement is unknown. The life expectancy of a population depends on a collection of environmental and socio-economic factors of the territory where they reside. Our aim was to compare the life expectancy of patients undergoing surgery for ascending aortic aneurysm with that of the general population matching by age, sex, and territory. In addition, we aimed to know the late complications, causes of death and risk factors. **Methods**: All patients who underwent elective replacement of an ascending aortic aneurysm at our institution between 2000 and 2019 were included. The long-term survival of the sample was compared with that of the general population using data of the National Institute of Statistics. **Results**: For patients who survived the postoperative period, observed cumulative survival at three, five and eight years was 94.07% (95% CI 91.87–95.70%), 89.96% (95% CI 86.92–92.33%) and 82.72% (95% CI 77.68–86.71%). Cumulative survival of the general population at three, five and eight years was 93.22%, 88.30%, and 80.27%. Cancer and cardiac failure were the main causes of death. **Conclusions**: Long-term survival of patients undergoing elective surgery for ascending aortic aneurysm who survive the postoperative period completely recover their life expectancy.

## 1. Introduction

After atherosclerosis, the aneurysm is the second most frequent disease of the aorta [1]. The current incidence of thoracic aortic aneurysm is approximately 8 in 100,000 patients per year [2].

Several environmental and genetic risk factors have been identified in its formation [3]. Once a segment of the aorta is aneurysmal, the entire aorta is considered as pathological [1]. Due to the risk of dissection and rupture, entities with extreme risk of immediate death, open elective surgery on the ascending aortic aneurysm is indicated when the diameter reaches certain limits. With the objective to reduce the aortic diameter, reduction aortoplasty and aortic wrapping are acceptable surgical techniques. Nevertheless, the most definitive solution is the replacement of the aortic aneurysm [4]. 

The patient’s life expectancy in the theoretical assumption of not having the aortic aneurysm plays a key role to decide if it is worth operating and what type of surgical technique is preferable. Physicians and surgeons usually consider that a patient’s life expectancy will be fully recovered after surgery. However, replacing a part of the aorta will not prevent the rest of it from being subject to the same risk factors that caused the aneurysmal formation. In addition, due to common risk factors, patients with aortic aneurysms have a higher risk of cardiovascular events than the general population [1,5]. Thus, even after a successful ascending aortic replacement, their life expectancy can be compromised. Therefore, any decision based on the theoretical recovery of that life expectancy can be made under false assumptions. 

Few studies have analysed the long-term follow-up of patients undergoing ascending aortic replacement. These studies are limited by the low number of patients [6,7,8], short follow-up [9,10] or high heterogeneity analysing at the same time patients with acute aortic syndrome and elective surgery for ascending aortic aneurysm [10,11].

Moreover, some studies [6,8,11,12,13] have described the long-term survival of patients undergoing ascending aortic surgery. But these results, without comparing them with the general population of the same territory, provide little information since the life expectancy of any group depends on a collection of environmental and socio-economic factors of the territory where they reside. The gross domestic product, the health system, food habits or the temperature are only some of the factors that have been shown to have an impact on the life expectancy of the general population [14]. In this line, there are significant differences among industrialized countries and even among regions of the same country. For example, in 2017, the life expectancy of a 65-year-old woman was 20.6 years in the USA and 24.4 years in Japan [15].

Our objective was to know if patients who undergo replacement of an ascending aortic aneurysm recover a life expectancy similar to that of the general population for the same age, sex, and territory. In addition, we aimed to know late complications, causes of death and the main risk factors in this population. 

## 2. Experimental Section

### 2.1. Sample and Data Collection

We included all patients who underwent elective replacement of an ascending aortic aneurysm at our institution between 2000 and 2019. Concomitant aortic valve or coronary surgeries were allowed. Aortic valve-sparing, Bentall-Bonno procedures and all surgeries on the aortic root or the aortic arch were included if the ascending aorta was also replaced. 

Patients were excluded if they underwent a previous surgery on the ascending aorta or the aortic root. Patients with acute aortic syndrome, chronic dissections, pseudoaneurysms or those who required concomitant mitral or tricuspid valve surgery were also excluded. 

All data relating to the pre-, intra-, and postoperative periods were collected retrospectively from a digital database completed prospectively by the patient’s surgeon. The postoperative period was taken as the first 30 days of follow-up, or until the date of hospital discharge if this was beyond 30 days. 

Data on death during follow-up were collected by one of the researchers who analysed the information in the medical records from all the health centres and hospitals of our Region. All hospitals and health centres of our region are connected via intranet so, from our institution, we could investigate all medical records and health reports. 

To compare the sample with the general population matched by age and sex, the tables of incidence of death provided by the National Institute of Statistics [16] for our region were used. This institute provides high-quality information on multiple statistics of the country. Regarding the incidence of death, the institute provides the information stratified by age, sex and regions of the nation. More information on the institute can be found in the Supplementary Material. 

### 2.2. Objectives

Primary objectives were: (1) to compare life expectancy and survival curves of patients who underwent replacement of an ascending aortic aneurysm with that of the general population matching for the same age, sex and territory; (2) to compare life expectancy and survival curves of those patients who survived the postoperative period and (3) to know their causes of death, risk factors for mortality and late complications. 

Secondary objectives were to compare the survival curves of these patients stratifying by bicuspid or tricuspid valve and by <70 and >70 years of age. 

### 2.3. Statistical Analysis

Quantitative and categorical variables were described as mean ± SD and *n* (%) respectively. 

To compare survival of the surgical sample with the general population matched by age and sex, the following estimations were calculated: (1) observed survival; (2) expected survival and (3) relative survival (RS).

Observed survival is the real survival of the surgical sample calculated by the usual Kaplan-Meier method.

Expected survival is the survival that a group of people from the general population would have if each individual was a copy of the same age, sex and region as the surgical sample. This is each individual of the general population being matched by age and sex with each individual of the surgical sample. 

This means that the expected survival is the survival that the surgical sample would have if they did not have the aortic aneurysm. Its calculation is performed by the Ederer II method, which is the calculation of the choice to know the expected survival of a sample [17]. To do that, we used the information on the incidence of death provided by the National Institute of Statistics for different ages, sex and region [16]. If the expected survival was included in the 95% confidence interval of the observed survival, no statistical differences were considered to exist. 

RS is an estimate of the survival that patients of the surgical sample would have in the theoretical assumption that they could only die from a problem associated with their aortic aneurysm [17,18]. Its calculation is made by the ratio between the observed survival rate and the expected survival rate. An SR of 100% in the first year would mean that all problems associated with the presence of an aortic aneurysm would have been completely solved with the replacement of the aneurysm. However, an RS of 80% in the first year would indicate that 20% (100–80%) of the patients would have died due to a problem derived from or associated with the aortic aneurysm [19]. Therefore, if the RS confidence interval includes 100%, there is no evidence that there is mortality associated with the aneurysm and suggests that the replacement has been completely effective in solving the problem [18].

One of the main advantages of the RS is that it allows knowing the mortality due exclusively to the disease under study, without knowing the causes of death [18].

To know the main risk factors for mortality, a Cox regression analysis was performed using as independent variables all factors that could influence the prognosis from a theoretical point of view. The proportionality of hazards assumption was tested by analysis of Schoenfield residuals. 95% CI was provided for Hazard Ratio (HR) and *p* values ≤0.05 were considered statistically significant. The variables of the model were chosen based on theoretical knowledge: age, sex, type 1 or type 2 diabetes, renal impairment, type of surgery (isolated replacement of the ascending aorta was the reference), chronic pulmonary disease, extracardiac arteriopathy, pulmonary hypertension, left ventricular dysfunction. 

All analyses were performed using STATA v.15.1 (STATA Corp, TX, USA). Observed survival, expected survival and the RS were calculated in an automated way using the “strs” command [20]. Using the previously described Ederer II method, this command allows, in an easy way, to match by sex and age.

Ethical approval was obtained from the corresponding IRB (reference number: 20/087).

## 3. Results

### 3.1. Patients, Type of Surgery and Postoperative Outcomes. 

There were 738 patients who underwent ascending aortic replacement due to aortic aneurysm. Of them, 232 (31.44%) were women and the mean age was 65.27 years ± 13.09. All patient characteristics are presented in Table 1. Three hundred and eighty-six patients (52.30%) underwent concomitant aortic valve replacement. One hundred and forty (18.97%) underwent aortic root remodelling with valve preservation. Eighty-six (11.65%) patients underwent isolated ascending aortic replacement and 30 (4.07%) individuals underwent ascending aorta and aortic arch replacement. All types of surgery are described in Table 2. Mean ascending aorta diameter was 50.93 ± 8.43 mm and 296 (40.11%) patients had a bicuspid aortic valve. 

Forty-four (5.96%) patients died during the postoperative period. Postoperative mortality for isolated ascending aortic surgery was 4 (4.65%), for aortic valve replacement and ascending aortic replacement was 20 (5.18%) and for concomitant aortic arch replacement was 8 (26.67%). Cardiopulmonary bypass time was 139.25 ± 60.62 minutes and aortic cross-clamping time was 112.27 ± 52.25 minutes. The median of hospital stay was 10 (8–14). Causes of death, postoperative complications and medications at discharge are shown in Table 2 and Table 3. All of the 144 (19.83%) patients who developed a new postoperative AF were treated with oral anticoagulants at discharge. There were 139 with vitamin K antagonists and 5 with novel oral anticoagulants.

### 3.2. Life Expectancy of the Whole Sample

There were no patients lost during follow-up. The mean follow-up for the censored individuals was 56.02 ± 36.37 months. There were 130 (17.61%) patients who died during the postoperative period and the follow-up. The main causes of death were shown in Table 3.

**Figure 1:**
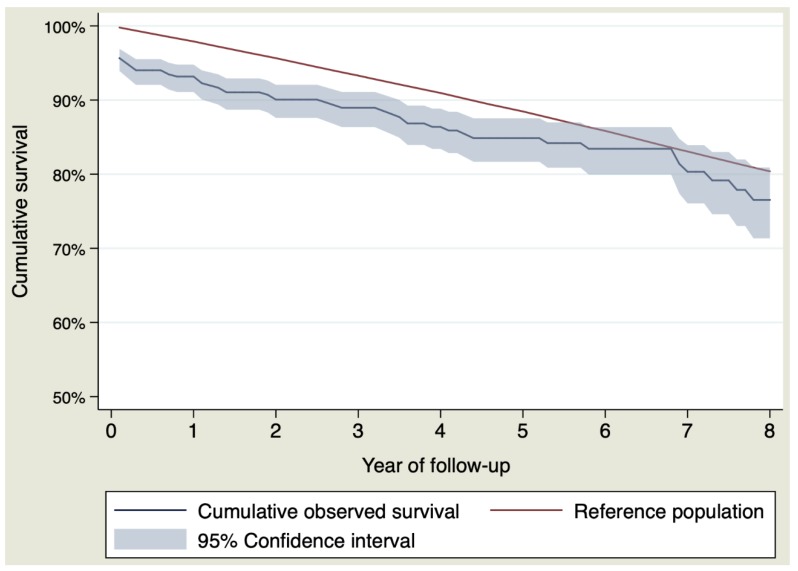
Observed and expected survival for the whole sample.

The observed survival of the sample was at 1, 3, 5 and 8 years of follow-up and was 93.17% (CI 95% 91.08–94.78%), 88.96% (CI 95% 86.35–91.10%), 84.86% (CI 95% 81.67–87.53%) and 76.53% (CI 95% 71.35–80.91%). Expected survival was at 1, 3, 5 and 8 years of follow-up, 97.90%, 93.30%, 88.46% and 80.39%. Table 4 shows the observed and expected survival for each year of follow-up and Figure 1 shows the survival curves. 

**Figure 2:**
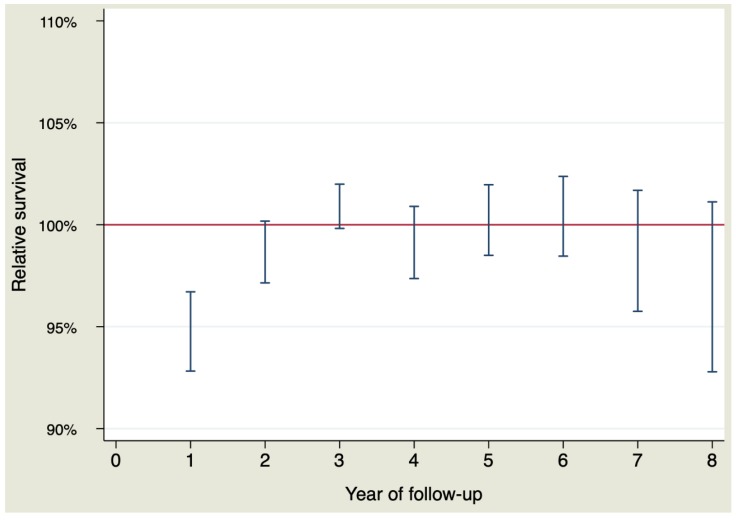
Relative survival for each year of follow-up.

The RS during the first year of follow-up showed anexcess of mortality due to the aneurysm RS = 95.02% (CI 95% 92.82–96.71%). The RS of the rest of the follow-up did not show an excess of mortality due to the disease, or what is the same, the expected survival was similar to the observed survival. Figure 2 and Table 4 show the RS by interval calculated for each year of follow-up.

### 3.3. Life Expectancy for Patients who Survive the Postoperative Period

**Figure 3:**
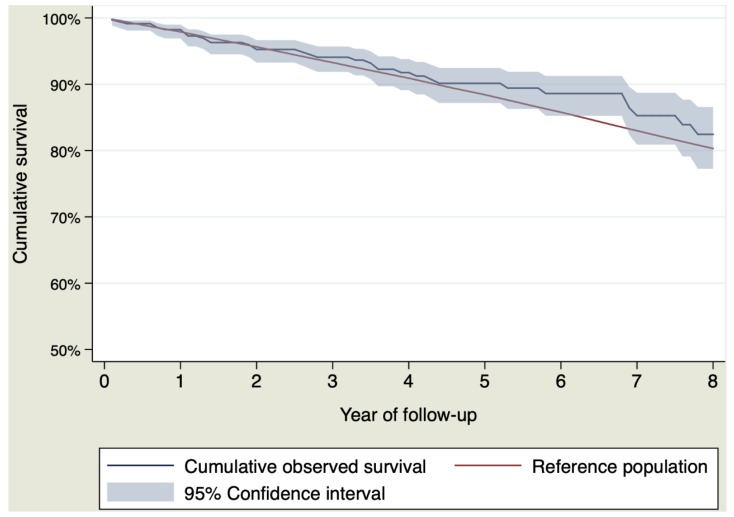
Observed and expected survival for patients who survived the postoperative period.

Among the 694 patients (94.04%) who survived the postoperative period, 86 (12.39%) patients died. Their observed cumulative survival at 1, 3, 5 and 8 years of follow-up was 98.29% (95% CI 96.85–98.97%), 94.07% (95% CI 91.87–95.70%), 89.96% (95% CI 86.92–92.33%) and 82.72% (95% CI 77.68–86.71%). The expected survival at 1, 3, 5 and 8 years of follow-up was 97.91%, 93.22%, 88.30% and 80.27%. Table 5 shows cumulative survival for the sample and reference population. Figure 3 shows the survival curves. 

The specific RS of the first year did not show an excess of mortality due to the aneurysm, 100.30% (CI 95% 98.92–101.09%). The rest of the calculated RS for each year of follow-up did not show mortality due to the aneurysm, or what is the same, the expected and observed mortality were similar. Table 5 shows the RS by interval for each of the years of follow-up. 

**Figure 4:**
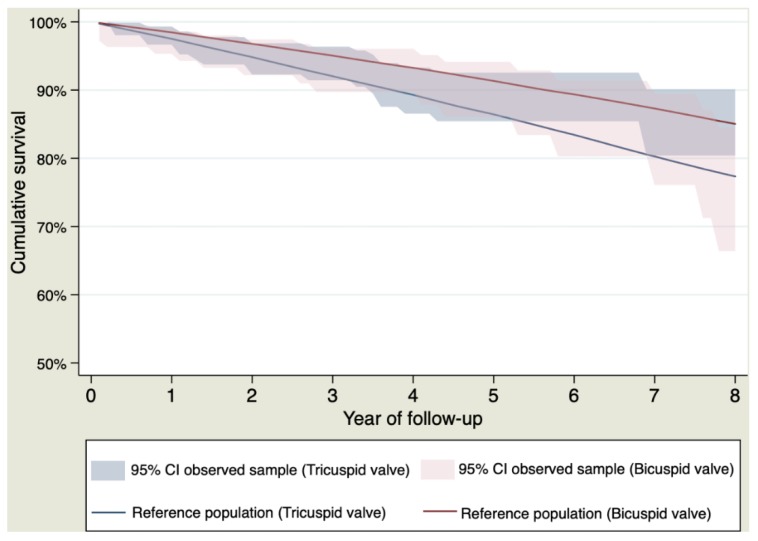
Survival curves stratified by bicuspid or tricuspid aortic valve for patients who survived the postoperative period.

**Figure 5:**
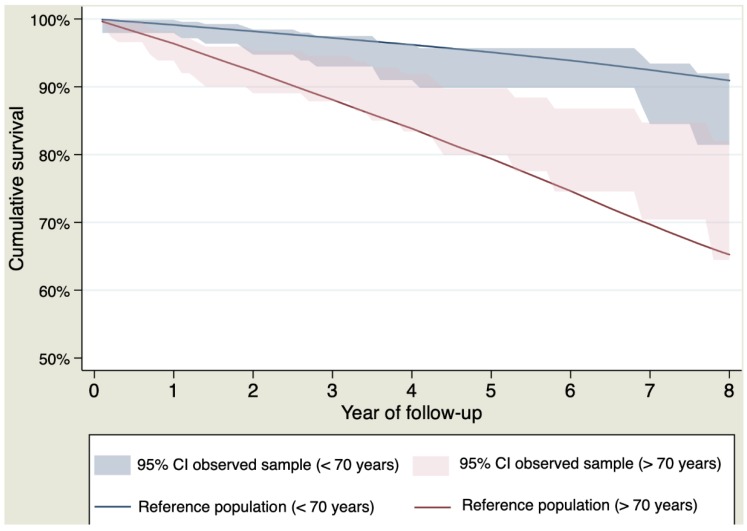
Survival curves stratified by age > or < 70 years for patients who survived the postoperative period.

Survival curves of the sample and the general population stratified by bicuspid or tricuspid valves and by age < or >70 years are shown in Figure 4 and Figure 5, respectively.

### 3.4. Causes of Death During the Follow-up, Risk Factors and Late Complications

In 718 patients (97.29%), the aorta did not require a second intervention. There were 20 patients who underwent surgery due to the aorta and 10 (1.36%) of them had endovascular surgery to treat another aneurysm in the descending aorta and another 10 (1.36%) patients required open aortic surgery. For eight of them, it was due to pseudoaneurysm and for two it was because of the presence of a new aneurysm in the aortic root. Thirty patients required cardiac surgery for other circumstances. The causes of re-operation can be consulted in Table 3.

After the Cox regression analysis, the following risk factors for mortality during the follow-up were identified: age (HR = 1.03 CI 95% 1.01–1.05; *p* = 0.002); two types of surgery, concomitant replacement of the aortic arch (HR = 4.95 CI 95% 1.94–12.60; *p* = 0.001) and concomitant replacement of the aortic arch and aortic valve (HR = 6.1 CI 95% 2.16–17.34; *p* = 0.001); and LVEF <20% (HR = 10.95 CI 95% 2.32–51.21; *p* = 0.002). Results of the Cox regression can be consulted in Table 6.

Among the 86 patients who died during the follow-up, cancer was the cause of death in 24 patients (27.90%), cardiac failure in 18 (20.93%) and the aorta only caused 2 confirmed deaths (2.32%) taking into account that 2 patients (2.32%) died from sudden death without autopsy. All causes of death are presented in Table 3.

## 4. Discussion

As the life expectancy of a population is greatly influenced by the geographical region where they live, we compared the life expectancy of patients who underwent ascending aortic replacement with that of the general population from the same region matched for age and sex. In addition, this study used for the first time the RS to know if these patients recovered their life expectancy after the operation. This method, common in studies on cancer therapies [17,18], has been recently used for the first time in the cardiovascular field [19] and allows us to calculate the risk of mortality due to the disease without knowing the causes of death [20].

Our main finding was that the life expectancy of patients who underwent replacement of an ascending aortic aneurysm and survived the postoperative period was similar to that of the general population. 

Analyzing the whole sample, that is, including patients who died during the postoperative period, patients who underwent replacement of the ascending aorta did not reach a life expectancy similar to that of the general population. This could be inferred from the lack of overlap of the CI of the observed survival curve with the expected survival curve. So, the likelihood of survival was lower in the surgical group than in the general population in the first six years and then equalized between the two groups from the beginning of the sixth year, remaining equal until the eighth year. This occurred because the RS, which is an estimation of the excess of mortality due to the disease (or associated conditions like surgery), was not the same throughout the whole follow-up period. The RS indicated an excess of mortality due to the aorta of 5% (100–95.02%) during the first year. Beyond this first year, the relative survival did not identify an excess of mortality due to the aorta in the rest of the follow-up. This was caused by perioperative mortality of almost 6%, which had a negative effect on survival in the surgical group. This observed postoperative mortality was higher than that predicted by the EuroSCORE II (3.68%) but less than that predicted by the logistic EuroSCORE (13.19%). Surgeries performed 20 years ago, when operative mortality was higher, can explain it. 

In the group of patients who survived the postoperative period, the survival curve was practically identical to that of the general population throughout the whole follow-up period. The RS by year of follow-up did not identify any year with an excess of mortality due to the disease indicating that the operation completely recovered their life expectancy. With perioperative mortality for isolated ascending aortic replacement of less than 1% reported in some recent studies [9,21], this finding gives an incredibly promising scenario from which we can infer that the aneurysm of the ascending aorta is nowadays a condition that does not have to affect long-term survival. In addition, the risk of a late complication associated with the aorta was very low (only 3 patients, 0.43%) indicating that the aorta is no longer a problem in these patients. Conversely, cancer and cardiac failure are the main causes of death during the follow-up, which reinforces the hypothesis that the problem of the aorta is solved.

Therefore, risk factors for aneurysm formation like hypertension or dyslipidemia were not strong enough to reduce life expectancy in these patients, which could be explained by a rigorous clinical follow-up after the surgery and throughout their life. 

In summary, patients with an ascending aortic aneurysm who undergo elective surgery to replace it and who survive the postoperative period can be informed that their life expectancy will be fully recovered. That life expectancy can be easily consulted in the corresponding national statistics.

This study has some limitations. First, it is subject to possible biases derived from its retrospective nature. Second, not all variables with a potential impact on late outcomes could be studied. Intraoperative or postoperative transfusion are examples. 

## 5. Conclusions

Long-term survival of patients undergoing elective surgery for ascending aortic aneurysm is fully conditioned by the operative mortality. Those who survive the postoperative period completely recover their life expectancy, which can be consulted in the corresponding national statistics. 

## Figures and Tables

**Table 1:** Patients characteristics.

Variable	Value
Age (years)	65.27 ± 13.09
Women	232 (31.44%)
Body mass index (kg/m^2^)	28.15 ± 4.54
Body surface area (m^2^)	1.85 ± 0.20
Hypertension	492 (66.67%)
Diabetes mellitus	
Type 1	12 (1.63%)
Type 2	65 (8.81%)
Dyslipidaemia	241 (32.66%)
Previous stroke	28 (3.79%)
Previous acute myocardial infarction	16 (2.27%)
Extracardiac arteriopathy	26 (3.53%)
Renal impairment	
Creatinine clearance >85 mL/min	520 (70.65%)
Creatinine clearance 50–85 mL/min	164 (22.28%)
Creatinine clearance <50 mL/min	52 (7.07%)
Chronic pulmonary disease	108 (14.63%)
Poor mobility	2 (0.27%)
EuroScore 2	3.68 ± 3.65
Logistic EuroSCORE	13.19 ± 9.86
NYHA functional class:	
NYHA I/IV	136 (18.43%)
NYHA II/IV	374 (50.68%)
NYHA III/IV	202 (27.37%)
NYHA IV/IV	26 (3.52%)
Previous atrial fibrillation	
Paroxysmal atrial fibrillation	34 (4.62%)
Persistent or permanent atrial fibrillation	115 (15.63%)
PASP	
31–55 mmHg	156 (21.14%)
> 55 mmHg	19 (2.57%)
LVEF (%)	
31–50%	164 (22.22%)
21–30%	29 (3.93%)
<20%	2 (0.27%)
Grade of aortic stenosis	
I	40 (5.42%)
II	29 (3.93%)
III	40 (5.42%)
IV	233 (31.57%)
Grade of aortic regurgitation	
I	74 (10.03%)
II	83 (11.25%)
III	134 (18.16%)
IV	232 (31.44%)
Bicuspid aortic valve	296 (40.11%)
Severe ventricular hypertrophy	111 (15.22%)
Diameter of the aorta (mm)	
Sinus of Valsalva	42.77 ± 7.07
Ascending aorta	50.93 ± 8.43
Aortic arch	40.6 ± 9.22

LVEF: Left ventricular ejection fraction; PASP: Pulmonary artery systolic pressure; NYHA: New York Heart Association.

**Table 2:** Characteristics of the operation.

Variable	Value
***Intraoperative characteristics***	
Type of Surgery Aortic valve replacement and ascending aorta replacement Aortic root remodelling with ascending aorta replacement Isolated ascending aorta replacement Bentall-Bonno procedures with ascending aorta replacement Ascending aorta and aortic arch replacement Ascending aorta replacement and aortic valve repair Aortic valve replacement, ascending aorta replacement and aortic arch replacement	386 (52.30%) 140 (18.97%) 86 (11.65%) 57 (7.72%) 30 (4.07%) 23 (3.11%) 16 (2.17%)
Cardiopulmonary bypass time	139 ± 60
Cross-clamping time	112 ± 52
Surgery with circulatory arrest	84 (11.38%)
Using deep hypothermia	13 (15.48%)
Using moderate hypothermia with antegrade cerebral perfusion	71 (84.52%)
Concomitant coronary surgery	114 (15.44%)
Number of the prosthetic tube	
26	75 (10.16%)
28	209 (28.32%)
30	340 (46.07%)
32	91 (12.33%)
34	21 (2.85%)
36	2 (0.27%)
EuroScore 2	3.68 ± 3.65
Logistic EuroSCORE	13.19 ± 9.86
***Postoperative complications***	
Permanent pacemaker	46 (6.23%)
New atrial fibrillation	144 (19.83%)
Reoperation for bleeding	48 (6.50%)
Stroke	33 (4.47%)
New renal failure	25 (3.38%)
***Medication at discharge***	
Angiotensin II receptor blockers	161 (21.82%)
Angiotensin-converting enzyme inhibitors	155 (20.00%)
Beta blockers	434 (59.78%)
Statins	278 (38.29%)

**Table 3:** Causes of re-operation and death.

Cause	Value
*Causes of re-operation*	
Aorta-related	
Treated by endovascular therapy	10 (1.36%)
Aneurysm	6 (0.81%)
Dissection	5 (0.54%)
Treated by open cardiac surgery	
Pseudoaneurysm	8 (1.08%)
New aneurysm	2 (0.27%)
Non aorta-related	
Endocarditis	10 (1.36%)
Prosthesis thrombosis	2 (0.36%)
Periprosthetic aortic regurgitation	3 (0.41%)
Prosthetic degeneration	3 (0.41%)
Myxoma	2 (0.36%)
Failed aortic repair	7 (0.95%)
***Causes of death***	
Peri-operative period *n* = 44	
Cardiogenic shock	21 (2.85%)
Hemorrhagic shock	6 (0.81%)
Infection/Sepsis	11 (1.49%)
Others	6 (0.81%)
Follow-up *n* = 86 (Between survivors of the postoperative period)	
Cancer	24 (3.46%)
Cardiac failure	18 (2.59%)
Infection or sepsis	10 (1.44%)
Stroke	6 (0.86%)
Acute aortic syndrome	3 (0.43%)
Sudden death	3 (0.43%)
Other cause	22 (3.17%)

**Table 4:** Cumulative survival of the sample and the reference population. Annual relative survival is also described for the whole sample.

Year of Follow-up	Cumulative Survival in the Sample	Cumulative Survival in the Reference	Annual Relative Survival*
First year	93.17% (CI 95% 91.08–94.78%)	97.90%	95.02% (CI 95% 92.82–96.71%)
Second year	90.06% (CI 95% 87.59–92.05%)	95.65%	98.98% (CI 95% 97.15–100.18%)
Third year	88.96% (CI 95% 86.35–91.10%)	93.30%	100.31% (CI 95% 99.82–101.99%)
Fourth year	86.37% (CI 95% 83.41–88.85%)	90.95%	99.60% (CI 95% 97.36–100.90%)
Fifth year	84.86% (CI 95% 81.67–87.53%)	88.46%	100.87% (CI 95% 98.50–101.96%)
Sixth year	83.42% (CI 95% 79.95–86.35%)	85.84%	101.29% (CI 95% 98.46–102.37%)
Seventh year	80.33% (CI 95% 76.05–83.92%)	83.06%	99.81% (CI 95% 95.75–101.69%)
Eighth year	76.53% (CI 95% 71.35–80.91%)	80.39%	98.46% (CI 95% 92.79–101.12%)

*Relative survival by interval. This is not a cumulative estimate.

**Table 5:** Cumulative survival of the sample and the reference population. Annual relative survival is also described. Data for survivors of the postoperative period.

Year of Follow-up	Cumulative Survival in the Sample	Cumulative Survival in the Reference	Annual Relative Survival*
First year	98.23% (CI 95% 96.91–98.99%)	97.90%	100.30% (CI 95% 98.92–101.09%)
Second year	95.24% (CI 95% 93.26–96.65%)	95.64%	99.31% (CI 95% 97.53–100.45%)
Third year	94.08% (CI 95% 91.87–95.70%)	93.27%	101.31% (CI 95% 99.81–101.99%)
Fourth year	91.76% (CI 95% 89.10–93.80%)	90.92%	100.10% (CI 95% 97.96–101.26%)
Fifth year	90.14% (CI 95% 87.17–92.46%)	88.42%	100.87% (CI 95% 98.48–101.96%)
Sixth year	88.61% (CI 95% 85.24–91.25%)	85.79%	101.29% (CI 95% 98.44–102.38%)
Seventh year	85.27% (CI 95% 80.90–88.72%)	83.00%	99.79% (CI 95% 95.68–101.69%)
Eighth year	82.45% (CI 95% 77.24–86.57%)	80.31%	100.07% (CI 95% 94.79–102.12%)

*Relative survival by interval. This is not a cumulative estimate.

**Table 6:** Results of the COX regression analysis showing the main risk factors for mortality.

Variable	HR	95% CI	*p* Value
Women	0.77	0.51–1.21	0.29
Age	1.03	1.01–1.05	0.002
Type of surgery			
Aortic valve replacement and ascending aorta replacement	1.62	0.86–3.03	0.14
Aortic root remodelling with ascending aorta replacement	1.19	0.49–2.86	0.74
Bentall-Bonno procedures with ascending aorta replacement	1.93	0.81–4.76	0.14
Ascending aorta and aortic arch replacement	4.95	1.94–12.6	0.001
Ascending aorta replacement and aortic valve repair	2.55	0.76–8.56	0.13
Aortic valve replacement, ascending aorta replacement and aortic arch replacement	6.1	2.16–17.34	0.001
Renal impairment			
Creatinine clearance 50–85 mL/min	1.39	0.90–2.17	0.14
Creatinine clearance <50 mL/min	1.73	0.98–3.07	0.059
Diabetes			
Type-2	0.89	0.45–1.72	0.85
Type-1	2.22	0.98–5.14	0.06
Extracardiac arteriopathy	0.33	0.08–1.37	0.13
Chronic pulmonary disease	1.2	0.74–1.95	0.46
PASP			
31–55 mmHg	0.99	0.25–4.12	0.99
>55 mmHg	1.21	0.78–1.88	0.39
LVEF (%)			
31–50%	0.88	0.55–1.42	0.61
21–30%	1.09	0.39–3.06	0.86
<20%	10.95	2.32–51.21	0.002

CI: Confidence Interval; HR: Hazard Ratio; LVEF: Left ventricular ejection fraction. PSAP: Pulmonary systolic artery pressure.

## Supplementary Materials

The following are available online at https://www.mdpi.com/2077-0383/9/3/615/s1.
