# Supplementary material for: Life Expectancy after Surgery for Ascending Aortic Aneurysm
Source: J Clin Med. 2020 Feb 25;9(3):615. doi: 10.3390/jcm9030615 (PMC7141111; doi:10.3390/jcm9030615)
Supplement: Supplementary file 1 [file jcm-09-00615-s001.pdf]

# SUPPLEMENTARY

# MATERIAL

The National Institute of Statistics is a legally independent institution affiliated with the Spanish Ministry of Economics and Business though the Secretary of State for Economics and Business Support. It is regulated by Law 12/1989 of 9 May on Public Statistical Function (LFEP, by its initials in Spanish), which regulates statistical activity for state purposes that is the exclusive remit of the State, and by the statute approved by Royal Decree 508/2001 of 11 May.

This law assigns to the National Institute of Statistics a fundamental central role in public statistical activity, expressly placing it in charge of large-scale official statistical operations such as demographic and economic censuses, demographic and social statistics, mortality rates, economic indicators, and compiling the electoral census (1). The information provided is therefore of the highest quality.

Spain is divided into *Autonomous Communities*. The Principality of Asturias is an *Autonomous Community* comprising a single *Region* in the northwest of Spain, with a population of slightly over one million. Our hospital, Hospital Universitario Central de Asturias, is a tertiary referral centre for multiple specialties.


1. Instituto Nacional de Estadística, [site in Internet]. Madrid, Spain. Available at: http://www.ine.es/ss/Satellite?L=es_ES&c=Page&cid=1254735910183&p=1254735910183&pagename=INE%2FINELayout
